# Effects and User-Reported Experiences of a Self-Management Mobile Health App for Grieving Adolescents: Randomized Controlled Trial

**DOI:** 10.2196/94777

**Published:** 2026-07-15

**Authors:** Rebecca Rhodin, Rakel Eklund, Anneli Silvén Hagström, Rolf Gjestad, Atle Dyregrov, Josefin Sveen

**Affiliations:** 1 Department of Women’s and Children’s Health, Uppsala University, Dag Hammarskjölds väg 14BUppsala, Sweden, 46 768031560; 2 Department of Social Work, Stockholm University, Stockholm, Sweden; 3 Center for Crisis Psychology, University of Bergen, Bergen, Vestland, Norway; 4 National Centre for Disaster Psychiatry, Department of Medical Sciences, Uppsala University, Uppsala, Sweden

**Keywords:** bereavement, grief, prolonged grief disorder, mobile health, digital mental health, self-management, cognitive behavioral therapy, youth, adolescents, mobile phone

## Abstract

**Background:**

Adolescents who experience the loss of a family member are at increased risk of adverse mental health outcomes, yet many face barriers or may be reluctant to access in-person or group-based support. mHealth (mobile health) interventions can help address these barriers by offering flexible, accessible, and low-threshold support.

**Objective:**

This study evaluated the short- and long-term mental health effects of Alba – Youth in Grief, a preventive self-management mobile app for bereaved adolescents. The primary outcome was symptoms of prolonged grief, while secondary outcomes included grief reactions, personal growth, and symptoms of posttraumatic stress and depression. User-reported helpfulness and negative experiences were also examined.

**Methods:**

In an unblinded randomized controlled trial (ClinicalTrials.gov NCT06093113), 126 adolescents aged 12‐19 years who had lost a parent or sibling were allocated to either the unguided Alba app (n=61) or an active control condition receiving unguided web-based psychoeducation (n=65). Online self-assessments were conducted at baseline and at 2, 6, and 12 months. Participants generally demonstrated high levels of distress at baseline, with 40% (50/126) reporting symptoms indicative of probable prolonged grief disorder according to *ICD-11* (*International Classification of Diseases, 11th Revision*) diagnostic scoring rules. Mental health outcomes were analyzed using linear mixed models to examine changes over time between groups, while user experiences were examined using descriptive statistics and summative content analysis.

**Results:**

Intention-to-treat analyses showed moderate reductions in prolonged grief symptoms at 12 months among adolescents randomized to Alba compared with the control group, with no significant effects at the 2- and 6-month follow-ups. The app group also demonstrated greater reductions in grief reactions, posttraumatic stress symptoms, and depressive symptoms compared with controls, with the strongest effects observed at long-term follow-up. No effect on personal growth was demonstrated. Most participants reported the app as helpful, while a minority disclosed negative experiences such as sadness.

**Conclusions:**

Overall, the findings indicate that Alba may be beneficial in reducing mental health symptoms among bereaved adolescents and highlight its potential as a safe, acceptable, and scalable mHealth intervention.

## Introduction

Losing a family member can be a profoundly disruptive event and may be particularly difficult during adolescence, as individuals strive to form an independent identity yet remain dependent on adults and family for support [1]. Most adolescents gradually adapt to life after loss, but some experience persistent high levels of grief that elevate the risk for mental health difficulties [2]. Compared with nonbereaved peers, bereaved adolescents face a two- to threefold risk of depression and an 8.6% higher risk of posttraumatic stress [3,4], alongside increased vulnerability to psychosocial problems such as substance use, self-harm, and suicidal ideation, as well as to premature death [5-7].

Additionally, some bereaved adolescents develop prolonged grief disorder (PGD), with prevalence estimates ranging from 10.4% to 32% [8]. Recently introduced into the *ICD-11* (*International Classification of Diseases, 11th Revision*) and the *DSM-5-TR* (*Diagnostic and Statistical Manual of Mental Disorders, Fifth Edition, Text Revision*), PGD is characterized by persistent yearning for the deceased, separation distress, and intense emotional pain causing functional impairment in adolescents, often manifesting in anger and protest behaviors [1,9]. Negative cognitions about the self, life, and the future, alongside maladaptive avoidance strategies (eg, avoiding loss reminders and daily activities), have been linked to more severe prolonged grief and posttraumatic stress symptoms [10].

Conversely, protective factors such as adaptive coping strategies, self-efficacy, emotional expression, communication, and social support can mitigate the aforementioned risks [8,11]. Having constructive ways of coping with loss may also promote positive psychological changes called posttraumatic growth, including strengthened relationships, personal resilience, and renewed appreciation of life [12]. Interventions that normalize loss, promote agency, and strengthen coping thus hold potential to reduce mental health risks and promote personal growth in bereaved adolescents [11-13].

Preventive interventions, suitable for adolescents regardless of symptom severity, are commonly delivered as leader-led support groups or grief camps and often involve parents as participants [14]. Many draw on cognitive behavioral therapy (CBT) principles [14], incorporating components such as psychoeducation, coping and emotion regulation training, and exercises for challenging negative thoughts [13,14]. Other programs apply the Sense of Coherence framework [15], which emphasizes making loss comprehensible, grief manageable, and fostering new meaning in life [16]. Some preventive interventions, such as the Family Bereavement Program, have demonstrated long-term mental health benefits up to 15 years postintervention [17]. Nonetheless, evidence generally suggests only small effects of preventive interventions on grief, and depressive and posttraumatic stress symptoms [14]. Moreover, methodological limitations, including small samples, limited follow-up, and reliance on completer data, underscore the need for more rigorous trials using intention-to-treat analyses and long-term follow-up [14].

Young grievers may also face barriers or reluctance to participate in family-based, in-person, or group formats [15,18]. mHealth (mobile health) technologies, delivering health interventions through smartphone applications, offer advantages such as reach, accessibility, and anonymity [19]. Although internet-delivered CBT interventions for bereaved adolescents exist, both unguided [20] and guided [21], no mHealth app has yet been made available to this group. However, stand-alone mHealth apps have reduced depressive symptoms in adolescents with moderate effects [22], and the My Grief app for bereaved parents demonstrated small to moderate reductions in prolonged grief and posttraumatic stress symptoms relative to a waitlist control [23,24]. Thus, although effect sizes for mHealth apps are typically modest, their accessibility and reach suggest they may represent a valuable complement to existing interventions.

The self-management mobile app Alba – Youth in Grief was co-developed with parentally bereaved adolescents recruited through a nonprofit organization offering grief support [25]. An internal pilot trial, constituting the first phase of this randomized controlled trial (RCT), confirmed the intervention’s overall feasibility. However, minor adjustments to recruitment and inclusion criteria were required [26]. The pilot also demonstrated acceptability, with participants reporting high satisfaction and Alba’s helpfulness in understanding grief, managing emotions, and supporting self-efficacy [26]. In addition, narratively structured interviews with participants included in the RCT study provided further insights into how the app contributed to meaningful changes in adolescents’ grief and daily lives. The findings indicated that coping strategies fostered a sense of control-, grief-, and emotion-tracking enhanced grief understanding and emotional expression, and psychoeducation normalized loss experiences and strengthened self-perception [27].

This study aimed to evaluate the effects of the Alba app on bereaved adolescents’ mental health over time. The primary objective was to assess the short-term effects of Alba on prolonged grief symptoms after 2 months of use, compared to an active control. Secondary objectives were to investigate its long-term effects on prolonged grief symptoms, as well as short- and long-term effects on grief reactions, personal growth, posttraumatic stress symptoms, and depressive symptoms over the first year of follow-up, compared to an active control. Another secondary aim was to assess user-reported helpfulness of, and negative experiences with, Alba.

## Methods

### Design

This study was a 2-armed RCT with an active control (ClinicalTrials.gov, identifier: NCT06093113). The intervention group received the Alba app, while the control group obtained psychoeducation via a website. This approach, using an active control, was implemented to account for attention and expectancy effects and to ensure all participants receive some form of assistance. Both groups received immediate access to their assigned intervention after randomization and completed online self-assessments at baseline and 2, 6, and 12 months. The first 36 participants of the RCT comprised the internal pilot study [26].

### Sample Size

A sample size of 124 was estimated to detect a moderate effect on the primary outcome (ie, prolonged grief) with 80% power at a 5% significance level, accounting for a projected 21% dropout. This target was retained despite the pilot study indicating a slightly higher dropout rate, as recruitment changes implemented after the pilot, such as advertising on adolescent-frequented social media platforms, were expected to reduce attrition [26].

### Participants

Eligibility criteria required participants to be aged 12‐19 years, bereaved of a parent and/or sibling at least one month before enrollment, have smartphone access, and understand Swedish. However, due to technical difficulties in downloading the app experienced by those aged 12 years in the pilot, the lower age limit was raised to 13 years for the remainder of the RCT [26]. There were no exclusion criteria.

### Procedure

Recruitment for this study took place between December 2023 and September 2024 through social media advertisements, collaborations with nonprofit organizations and Swedish regions and municipalities, which shared study information within their networks and digital channels. Interested adolescents were directed to this study’s website for information and a digital sign-up form. A researcher then screened for eligibility via telephone or text message. For participants aged younger than 15 years, guardian contact information was collected, and verbal consent was obtained from all legal guardians in accordance with Swedish law.

Eligible participants then received a link via email or text message to a digital consent form hosted in REDCap (Vanderbilt University) [28,29]. After providing consent, participants completed baseline assessments and were then randomized. Following randomization (described under the Randomization section), participants received the Alba app (iOS or Android) or the control website by email and were free to use them as needed, with no recommended frequency or pattern of use. A researcher followed up one week later to confirm access for all participants. Follow-up assessments were conducted at 2, 6, and 12 months. Participants received up to 3 reminders by email and/or text message for each assessment.

### Randomization

After completing the baseline assessment, participants were randomized (1:1) to the intervention or control condition using REDCap [30]. The randomization sequence was generated by an external statistician using an unstratified block design of 20 allocations. The sequence was uploaded into REDCap by the first author without review, ensuring allocation concealment at the point of assignment. Once randomized, allocations were automatically revealed to the researcher but could not be altered, and participants were notified of their assigned condition via email and/or text message. Thus, neither participants nor researchers were blinded to conditions.

To prevent cross-group contamination, where participants access the intervention not assigned to them, siblings enrolling in this study were nonrandomly allocated to the same condition (n=5). Siblings were identified through comparison of shared residential addresses, and when identified, the later-enrolled siblings were assigned to the same condition as the first. No further stratification procedures were applied.

### The App Alba – Youth in Grief

Alba – Youth in Grief is a stand-alone self-management app designed to support bereaved adolescents by strengthening coping, communication, and social support, while addressing cognitive, emotional, and behavioral aspects of grief. Its content is based on CBT principles and the Sense of Coherence framework [16]. The app was co-developed with 6 parentally bereaved adolescents, together with researchers and an advisory group, to ensure age-appropriate functions, an appealing design, and language tailored for users aged 12‐19 years, as described previously [25]. Texts are intentionally brief to enhance readability, and interactive elements, such as an avatar guide, active navigation choices, and audio with matching text for breathing- and mindfulness exercises, are included.

Built on the structure of the My Grief app for bereaved parents [23,31,32] and PTSD (posttraumatic stress disorder) Coach [33,34], Alba is organized into 5 sections that can be accessed freely according to users’ individual needs and preferences, rather than in a predefined order:

- What is grief?: psychoeducation on grief and common reactions to foster understanding of grief and normalize experiences.
- What can I do?: practical tools (eg, mindfulness, breathing, and writing) for emotion regulation, processing of loss, and memorializing the deceased.
- How am I feeling?: emotion and grief intensity tracking features, with optional daily notifications, to support self-awareness.
- How do I seek support?: guidance on help-seeking and communicating grief-related needs.
- The private part: a memory bank for the deceased and a personalized safety plan to aid problem-solving in times of distress.

### Control Intervention

The control intervention was delivered through a password-protected section of this study’s website, to which participants received unlimited access by email after randomization. It contained abbreviated versions of about one-third of the psychoeducational texts from Alba’s “What is grief?” section, covering emotions, thoughts, behaviors, communication, and grief within the family. Unlike in the app, these texts included no advice or exercises for managing grief reactions. The website also provided contact details for 2 support services, including 1 emergency resource, and 1 nonprofit organization for individuals with suicidal ideation, representing roughly one-tenth of the support links available in the app. No other Alba content was included.

### Measurements

#### Overview

The primary outcome was symptoms of prolonged grief, with secondary outcomes of grief reactions, personal growth, and symptoms of posttraumatic stress and depression. In this study, prolonged grief symptoms refer to reactions associated with the diagnostic criteria for PGD, whereas grief reactions refer to common grief-related responses not tied to any specific diagnosis. All mental health outcomes were measured at baseline and at 2, 6, and 12 months.

#### Sociodemographic and Loss-Related Information

The baseline assessment included a self-constructed questionnaire, which collected information on sociodemographic characteristics (eg, gender, age, residential area, and country of birth) and loss-related information (eg, time since death, relationship to deceased, and cause of death).

#### Prolonged Grief

Prolonged grief symptoms were assessed with the Traumatic Grief Inventory-Kids-Self Report+ (TGI-K-SR+) [35,36]. The measure consists of 16 items rated from 1 (“never”) to 5 (“always”), where respondents are asked to indicate the frequency of experienced reactions during the last month as a consequence of the death. Items, for instance, include “I have felt guilty of the death of [deceased]” and “I have had trouble accepting that [deceased] is dead.” The measurement is designed in accordance with both *DSM-5-TR* and *ICD-11* symptom criteria for PGD and yields a total score of 16‐80. While optimal cutoff scores for probable PGD have been estimated in the initial validation of the instrument, these are advised to be used cautiously, and scoring rules may be more accurately used [37]. Scoring rules for *DSM-5-TR* require endorsement of ≥1 B-criterion symptom (items 1‐2), ≥3 of the C criterion symptoms (items 3‐11), and the D criterion symptom (item 16), while the liberal scoring rule for *ICD-11* specified the need for ≥1 B criterion symptom (items 1‐2), ≥1 C criterion symptom (items 3‐4, 6‐9, and 12‐15), and the E criterion symptom (item 16) to be endorsed. Previous work has shown strong internal consistency and good convergent validity for related versions [37,38]. In the present study, internal consistency was excellent at baseline (total omega *Ω*=.91). As the TGI-K-SR+ was not previously available in Swedish, the measure was translated from English and culturally adapted following recommended procedures [39], including forward-backward translation and pretesting with bereaved adolescents. For a detailed description, see Rhodin et al [26].

#### Grief Reactions and Personal Growth

Grief reactions and personal growth were measured using the Hogan Inventory for Bereavement-Short Form for Children and Adolescents (HIBSF-CA) [40]. The inventory includes 21 items scored from 1 (“does not describe me at all”) to 5 (“describes me very well”), forming 2 independent subscales: grief (10 items, range 10‐50) and personal growth (11 items, range 11‐55). Items assess the extent of grief-related thoughts and emotions experienced during the last 2 weeks, with higher scores indicating more severe grief reactions or greater personal growth, respectively. Example items include “I don’t think I will ever be happy again” (grief) and “I am more aware of others’ feelings” (personal growth). The personal growth subscale is conceptually aligned with posttraumatic growth as described in the broader literature; accordingly, findings are discussed in relation to previous research on posttraumatic growth.

Reliability of the HIBSF-CA has been shown to be strong [40], and internal consistency in this study was good at baseline (grief *Ω*=0.85; personal growth *Ω*=0.90). The HIBSF-CA was translated into Swedish and culturally adapted using the same procedures as the TGI-K-SR+ [26,39].

#### Posttraumatic Stress

Posttraumatic stress symptoms were assessed with the Child PTSD Symptom Scale–Self-Report Version for *DSM-5* (*Diagnostic and Statistical Manual of Mental Disorders, Fifth Edition*) [41]. The measure consists of 20 symptom items (range 0‐80) rated on a 5-point scale from 0 (“not at all”) to 4 (“almost always”), plus 7 items assessing impairment in daily life. Symptom items assess how frequently the respondent has experienced a symptom during the last month, for example, “being jumpy or easily scared,” and a cutoff score of 31 has been suggested to indicate probable PTSD [41]. The inventory has shown strong psychometric properties in adolescents [41] and has demonstrated high internal consistency with bereaved adolescents in a previous Swedish study [42]. It showed excellent internal consistency in this study at the baseline assessment (*Ω*=.92).

#### Depression

Depressive symptoms were measured with the Patient Health Questionnaire–9 [43], consisting of 9 items scored from 0 (“not at all”) to 3 (“almost every day”; range 0‐27), where the respondent is asked to indicate how often they have been troubled by a symptom during the last 2 weeks. Example items include “little interest or pleasure in doing things” and “feeling down, depressed, or hopeless.” Cutoffs of ≥10 and ≥15 have been suggested for moderate and severe depression, respectively. The Patient Health Questionnaire–9, previously used with adolescents [44, 45], demonstrated high reliability in Swedish samples [46] and showed good internal consistency at baseline in this study (Ω = .86).

#### App Helpfulness and Negative Experiences

At the 2-month follow-up, participants in the intervention group rated the app’s perceived helpfulness on 9 items scored from 1 (“no, not at all”) to 4 (“yes, completely”). Items addressed Alba’s usefulness for understanding grief reactions, regulating emotions, promoting self-efficacy, supporting grief communication, and facilitating help-seeking. Potential negative experiences were assessed using the yes or no item: “did you experience any negative consequences from using the app (for example, feeling sad or unwell)?” Participants answering “yes” were invited to provide further details in a free-text response. Most evaluation questions were adapted from the My Grief trial [32] and earlier intervention studies [33,47]. Additional evaluation items on self-reported app use were collected, and objective app engagement data were collected throughout the current study using Google Analytics. As usage data were anonymous and available only at the aggregate group level, they were used to describe overall app engagement but did not permit analyses of associations between individual app use and mental health outcomes.

### Statistical Analysis

All analyses were conducted in R software (version 2025.09.2+418; R Foundation). To examine the intervention’s short- and long-term effects on primary and secondary outcomes, linear mixed models (LMMs) were applied (*lme4* package 1.1‐37; *lmerTest* package 3.1‐3). The internal consistency of all measurements was assessed before analysis using McDonald total ω to evaluate their psychometric reliability at baseline (*psych* package 2.5.6). Missing data were assumed missing at random, with no imputation used. Assumptions of normality and homoscedasticity of residuals were checked in connection with the analysis (performance package 0.15.2).

Intention-to-treat analyses were performed, including all randomized participants, with no exclusions made from the analytic sample due to missing follow-up data. For each outcome, a separate LMM was estimated incorporating all time points and fixed effects of time, condition, and their interaction. These random-intercept models with fixed slopes used baseline as the reference time point, such that time effects reflected change from baseline and interaction terms represented differential change between groups. In addition, sensitivity analyses using complete cases were performed to validate the robustness of the findings, with sample sizes ranging from 74 to 77 participants depending on the outcome. Between-group effect sizes (Cohen’s *d*) with 95% CIs were further estimated using change scores from baseline to all follow-ups for each outcome (*effsize* package, version 0.8.1; *dplyr* package, version 1.1.4). Independent-samples *t* tests were performed on baseline outcome measures to compare participants who did and did not complete all assessments, both between study groups and within each group separately.

Items on app helpfulness and the proportion of participants reporting negative experiences were analyzed using descriptive statistics. Free-text responses describing negative experiences were examined using summative content analysis [48]. This approach enabled systematic identification and quantification of words and expressions related to adverse events. The analytical process entailed relevant terms being identified, grouped into categories, and quantified to classify different types of negative experiences. Furthermore, descriptive statistics on objective app engagement were retrieved from Google Analytics by filtering the period from the first participant receiving access to Alba until the last participant completed the 2-month follow-up assessment, thereby corresponding to the time period covered by the app evaluation survey.

### Ethical Considerations

This study was approved by the Swedish Ethical Review Authority (No. 2023‐0430901). Research with bereaved adolescents raises specific ethical concerns; according to previous research, primarily relating to informed consent, safeguarding confidentiality, and balancing potential risks and benefits [49]. In relation to supporting participants’ autonomy, study information was tailored to be age-appropriate and in a clear, accessible format, and informed consent was obtained for all participants. Participation was continuously emphasized as voluntary, and adolescents were reminded they could withdraw at any time without explanation and without affecting access to the intervention. Participants received no compensation. To ensure participant privacy and confidentiality, data was collected using REDCap and subsequently transferred to secure servers hosted by Uppsala University, and stored separately from code keys with access restricted to authorized personnel.

Although researching sensitive topics inevitably carries a risk of distress, many adolescents have valued research participation as an opportunity to use their experiences for positive purposes [50,51]. To mitigate distress concerns, control participants received the app after trial completion. Participants’ well-being was further safeguarded by monitoring suicidal ideation during assessments. If any indication of suicidal thoughts emerged, a researcher contacted the adolescent to assess severity and offered guidance on how to access appropriate professional support.

## Results

### Sample Characteristics

Of 126 included adolescents, 83% (104/126) of adolescents identified as female, and 93% (117/126) of adolescents were born in Sweden, with an average age of 16 (SD 1.93; range 12‐19) years. Regarding loss experiences, 43% (54/126) of participants had lost fathers, and 65% (82/126) of participants had lost a family member due to disease. The mean time since loss was approximately 3 years (SD 3.85; range 0.12‐22.97). More information on demographics and loss-related information for both the intervention group and the control group can be found in Table 1.

At baseline, 33% (42/126) vs 40% (50/126) of participants reported symptoms indicative of probable PGD using the diagnostic scoring rule for *DSM-5-TR* and *ICD-11*, respectively. Additionally, 25% (31/126) of participants scored above the cutoff for moderate depression in the self-reports and 28% (35/126) for severe depression, with 40% (51/126) exceeding the threshold for probable PTSD. Overall, 63% (80/126) vs 66% (83/126) of participants reported symptoms indicating the presence of at least one of the diagnoses when using *DSM-5-TR* and *ICD-11* scoring rules for PGD, respectively. No formal statistical comparison was conducted between groups, as any observed differences were assumed to reflect chance variation following randomization. However, inspection of descriptive statistics indicated comparable demographic characteristics and baseline symptom levels, with slightly higher posttraumatic stress symptoms in the intervention group (Table 2)

**Table 1.** Demographic characteristics for adolescents completing baseline assessment (N=126).

Demographic characteristics	Intervention group (n=61)	Control group (n=65)	Total sample (N=126)
Age (years)			
Mean (SD)	16.0 (1.8)	15.7 (2.0)	15.9 (1.9)
Range	12.0-19.0	12.0-19.0	12.0-19.0
Sex, n (%)			
Female	53 (86.9)	51 (78.5)	104 (82.5)
Male	7 (11.5)	13 (20.0)	20 (15.9)
Other	1 (1.6)	1 (1.5)	2 (1.6)
Residential area, n (%)			
Countryside or small town	18 (29.5)	24 (36.9)	42 (33.3)
Small or medium-sized city	31 (50.8)	19 (29.2)	50 (39.7)
Large city	12 (19.7)	22 (33.9)	34 (27.0)
Country of birth, n (%)			
Sweden	56 (91.8)	61 (93.9)	117 (92.9)
Nordic	2 (3.3)	1 (1.5)	3 (2.4)
Europe	1 (1.6)	2 (3.1)	3 (2.4)
Outside of Europe	2 (3.3)	1 (1.5)	3 (2.4)
Years since death			
Mean (SD)	3.0 (3.6)	3.6 (4.1)	3.3 (3.9)
Range	0.2-15.2	0.1-23.0^a^	0.1-23.0^a^
Family member deceased, n (%)			
Mother	11 (18.0)	17 (26.2)	28 (22.2)
Father	25 (41.0)	29 (44.6)	54 (42.9)
Brother	16 (26.2)	12 (18.5)	28 (22.2)
Sister	9 (14.8)	8 (12.3)	17 (13.5)
Other^b^	2 (3.3)	2 (3.1)	4 (3.2)
Type of death, n (%)			
Disease	37 (60.7)	45 (69.2)	82 (65.1)
Accident/murder/another act of violence	13 (21.3)	7 (10.8)	20 (15.9)
Suicide	9 (14.8)	12 (18.5)	21 (16.7)
Other^c^	3 (4.9)	3 (4.6)	6 (4.8)

a Including one participant who had lost a sibling before their own birth.

b Including stepmother, stepfather, or grandfather with parental role.

c Including overdose, pregnancy loss, and death attributed to medical complications caused by hospital error.

**Table 2.** Self-reported symptom levels at baseline (T0; n=126), 2 months (T1; n=94), 6 months (T2; n=87), and 12 months (T3; n=84), and between-group effect sizes based on change from baseline^a^.

	Intervention group, mean (SD)	Control group, mean (SD)	Between-group, *d* (95% CI)
Prolonged grief			
Baseline	51.26 (12.35)	50.09 (9.47)	—^b^
2 months	44.40 (11.34)	47.11 (11.00)	0.45 (0.01 to 0.88)
6 months	42.80 (11.38)	43.67 (11.98)	0.25 (−0.17 to 0.67)
12 months	38.76 (10.98)	43.89 (11.68)	0.64 (0.18 to 1.10)
Grief			
Baseline	27.31 (8.11)	25.54 (7.24)	—
2 months	24.84 (6.67)	25.53 (7.28)	0.49 (0.04 to 0.93)
6 months	25.05 (7.04)	23.86 (6.85)	0.11 (−0.31 to 0.55)
12 months	22.12 (6.69)	24.63 (6.81)	0.68 (0.22 to 1.15)
Personal growth			
Baseline	31.69 (8.76)	33.39 (8.00)	—
2 months	35.09 (8.81)	33.55 (7.87)	−0.27 (−0.71 to 0.18)
6 months	36.54 (9.24)	34.89 (8.12)	−0.29 (−0.72 to 0.14)
12 months	36.34 (10.65)	34.37 (8.67)	−0.32 (−0.77 to 0.14)
Posttraumatic stress			
Baseline	30.82 (16.73)	25.54 (12.48)	—
2 months	23.88 (13.33)	26.55 (14.65)	0.58 (0.14 to 1.03)
6 months	22.84 (13.98)	23.21 (14.62)	0.29 (−0.14 to 0.72)
12 months	15.93 (12.47)	22.70 (14.85)	0.70 (0.24 to 1.17)
Depression			
Baseline	11.57 (6.65)	9.39 (4.83)	—
2 months	8.77 (5.41)	9.68 (5.43)	0.45 (0.01 to 0.89)
6 months	9.21 (5.37)	8.46 (4.18)	0.12 (−0.31 to 0.54)
12 months	7.29 (5.07)	8.71 (5.56)	0.34 (−0.11 to 0.79)

a Positive *d* values indicate greater reductions in outcome scores in the intervention group relative to the control group, whereas negative values indicate greater increases in the intervention group relative to the control group.

b Not applicable.

### Dropout Analyses

**Figure 1.**
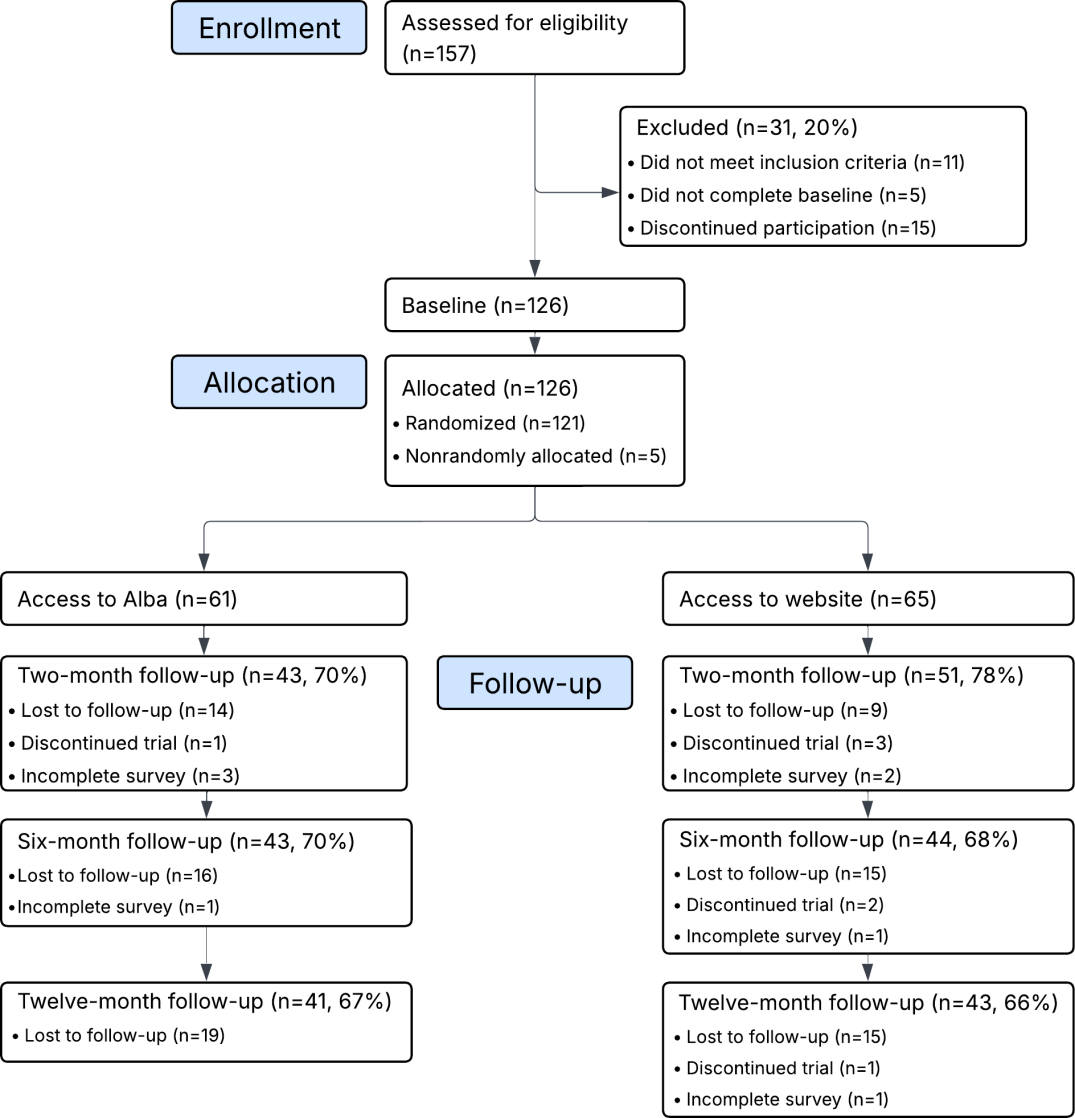
Flowchart of participants.

Overall, 24 participants completed only the baseline assessment and did not contribute follow-up data (see Figure 1 for participant flow). Baseline comparisons (Tables S1-S3 in Multimedia Appendix 1) showed that noncompleters in the intervention group (n=23) reported higher baseline posttraumatic stress and depressive symptoms than noncompleters in the control group (n=26), while intervention-group completers (n=38) reported higher personal growth than noncompleters (n=23), with no other baseline differences observed.

### Primary Outcome

**Figure 2.**
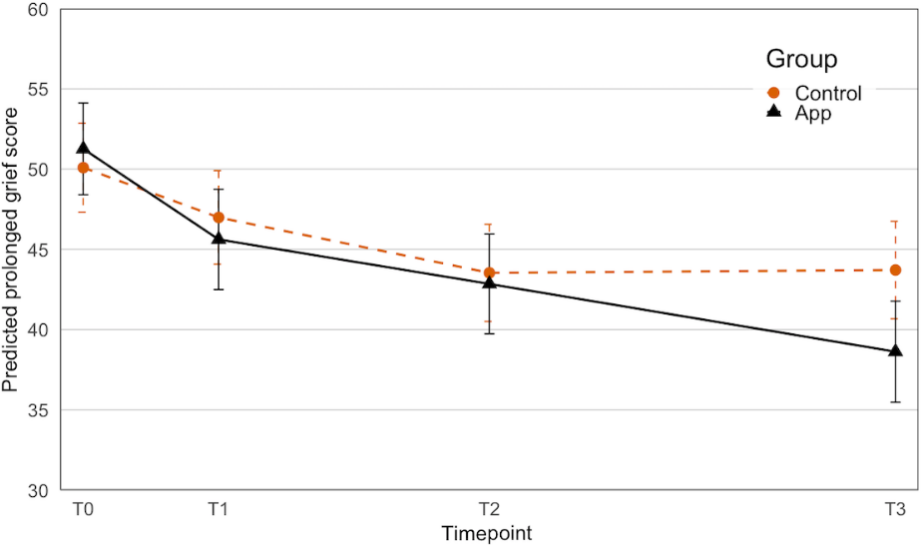
Predicted mean prolonged grief score (TGI-K-SR+) for the app and control groups across assessment points (T0=baseline, T1=2 months, T2=6 months, and T3=12 months), displayed using proportional spacing to reflect actual time intervals. Values represent estimated marginal means from the LMM, with 95% CIs displayed as error bars. LMM: linear mixed model; TGI-K-SR+: Traumatic Grief Inventory–Kids–Self Report+.

The intention-to-treat analysis revealed no statistically significant condition-by-time interaction for prolonged grief at the 2-month follow-up, although the corresponding between-group effect size suggested a small-to-moderate effect (Cohen’s *d*=0.45, 95% CI 0.01 to 0.88), or the 6-month follow-up (*d*=0.25, CI −0.17 to 0.67). However, the app group exhibited significantly greater symptom reductions than the control group at the 12-month follow-up (see Figure 2 and Table 3), demonstrating moderate between-group effect sizes (*d*=0.64, CI 0.18 to 1.10; Table 2).

**Table 3.** Intention-to-treat LMM^a^ results for primary and secondary outcomes (n=126). Model estimates represent changes from baseline (T0) and group differences relative to the control group reference category.

Outcome and fixed effect	Estimate (β)	SE	95% CI	*P* value
Prolonged grief				
Intercept	50.09	1.40	47.35 to 52.83	<.001
Condition	1.17	2.02	−2.77 to 5.11	.56
Time (T0-T1)	−3.10	1.16	−5.37 to −0.84	.008
Time (T0-T2)	−6.56	1.23	−8.96 to −4.16	<.001
Time (T0-T3)	−6.38	1.24	−8.80 to −3.96	<.001
Condition x T0-T1	−2.54	1.73	−5.90 to 0.82	.14
Condition x T0-T2	−1.86	1.77	−5.30 to 1.58	.29
Condition x T0-T3	−6.26	1.80	−9.76 to −2.77	<.001
Grief				
Intercept	25.54	.93	23.73 to 27.35	<.001
Condition	1.77	1.33	−.83 to 4.37	.19
Time (T0-T1)	−.08	.76	−1.57 to 1.40	.91
Time (T0-T2)	−1.85	.81	−3.42 to −0.28	.02
Time (T0-T3)	−.75	.81	−2.33 to 0.83	.36
Condition x T0-T1	−2.22	1.12	−4.41 to −0.03	.049
Condition x T0-T2	−.60	1.15	−2.85 to 1.64	.60
Condition x T0-T3	−4.75	1.17	−7.03 to −2.48	<.001
Personal growth				
Intercept	33.38	1.09	31.26 to 35.51	<.001
Condition	−1.70	1.56	−4.75 to 1.35	.28
Time (T0-T1)	.24	.90	−1.52 to 1.99	.79
Time (T0-T2)	1.09	.95	−.76 to 2.94	.25
Time (T0-T3)	.57	.96	−1.30 to 2.44	.55
Condition x T0-T1	1.49	1.33	−1.10 to 4.08	.26
Condition x T0-T2	2.14	1.36	−.50 to 4.80	.12
Condition x T0-T3	2.67	1.38	−.01 to 5.36	.05
Posttraumatic stress				
Intercept	25.54	1.82	21.98 to 29.09	<.001
Condition	5.28	2.62	.17 to 10.39	.045
Time (T0-T1)	.79	1.47	−2.08 to 3.66	.59
Time (T0-T2)	−3.34	1.56	−6.37 to −0.31	.03
Time (T0-T3)	−2.75	1.57	−5.80 to 0.30	.08
Condition x T0-T1	−6.02	2.17	−10.25 to −1.79	.006
Condition x T0-T2	−3.79	2.23	−8.13 to 0.54	.09
Condition x T0-T3	−10.86	2.26	−15.25 to −6.47	<.001
Depression				
Intercept	9.38	.68	8.05 to 10.72	<.001
Condition	2.19	.98	.27 to 4.11	.03
Time (T0-T1)	−.06	.61	−1.24 to 1.12	.93
Time (T0-T2)	−1.27	.65	−2.53 to −0.00	.05
Time (T0-T3)	−1.09	.65	−2.35 to 0.17	.09
Condition x T0-T1	−2.14	.90	−3.90 to −0.39	.02
Condition x T0-T2	−.85	.92	−2.65 to 0.95	.36
Condition x T0-T3	−2.83	.94	−4.66 to −1.01	.003

a LMM: linear mixed model.

### Secondary Outcomes

**Figure 3.**
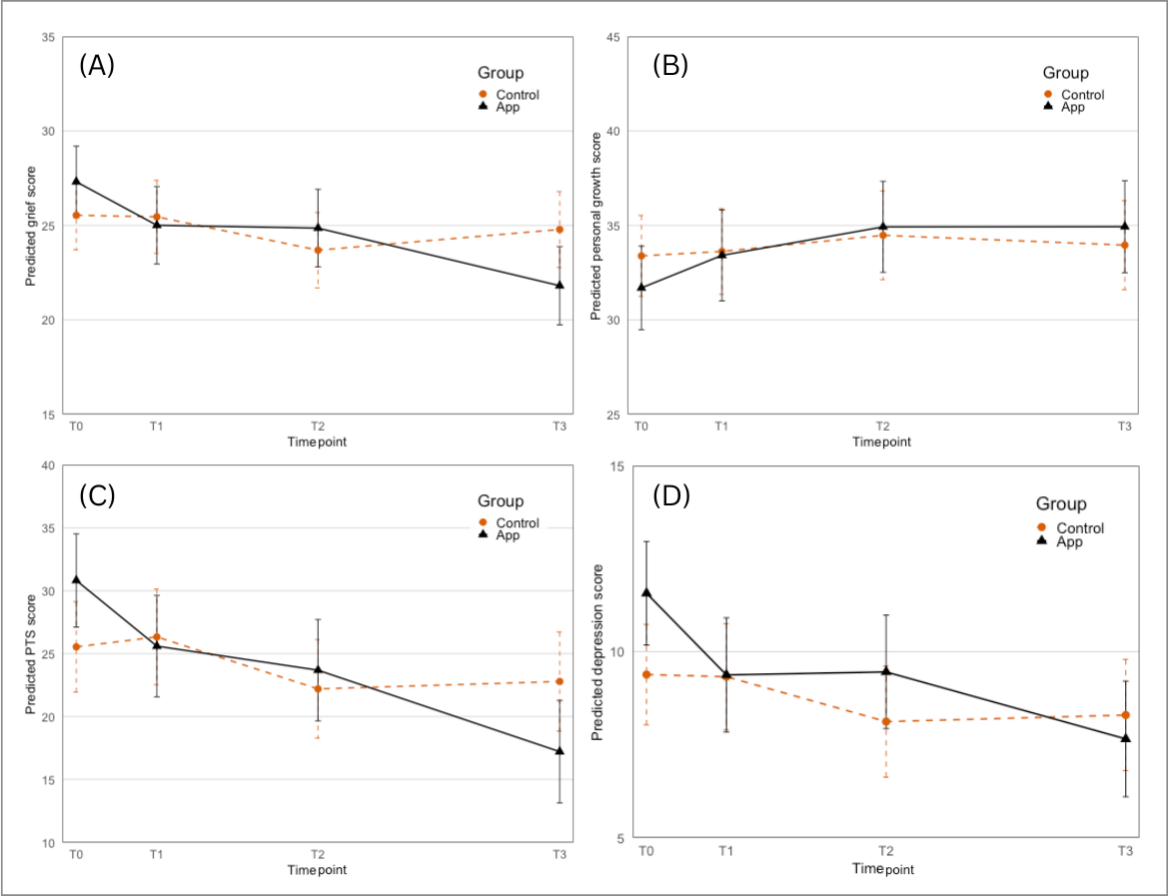
Predicted mean (A) grief (HIBSF-CA), (B) personal growth (HIBSF-CA), (C) posttraumatic stress (CPSS-5-SR), and (D) depression (PHQ-9) scores for the app and control groups across assessment points (T0=baseline, T1=2 months, T2=6 months, and T3=12 months). Time points are displayed using proportional spacing to reflect actual time intervals. Values represent estimated marginal means from the LMM, with 95% CIs shown as error bars. CPSS-5-SR: Child PTSD (posttraumatic stress disorder) Symptom Scale–Self-Report Version for *DSM-5* (*Diagnostic and Statistical Manual of Mental Disorders, Fifth Edition*); HIBSF-CA: Hogan Inventory for Bereavement–Short Form for Children and Adolescents; LMM: linear mixed model; PHQ-9: Patient Health Questionnaire–9; PTS: posttraumatic stress.

The app group demonstrated a steeper decline in grief reactions as well as posttraumatic stress and depressive symptoms compared to the control group, although only the differences at 2 months and 12 months reached statistical significance (Table 3 and Figure 3).

Effect sizes based on change from baseline supported this pattern, with the largest between-group differences for grief reactions (T1: *d*=0.49, CI 0.04 to 0.93; T2: *d*=0.11, CI −0.31 to 0.55; T3: *d*=0.68, CI 0.22 to 1.15) and posttraumatic stress (T1: *d*=0.58, CI 0.14 to 1.03; T2: *d*=0.29, CI −0.14 to 0.72; T3: *d*=0.70, CI 0.24 to 1.17) emerging at the last follow-up and demonstrating moderately sized effects of the app (Table 2). For depressive symptoms, the effect size was small (T1: *d*=0.45, CI 0.01 to 0.89; T2: *d*=0.12, CI −0.31 to 0.54; T3: *d*=0.34, CI −0.11 to 0.79). Analysis yielded no statistically significant intervention effect on personal growth (T1: *d*=−0.27, CI −0.71 to 0.18; T2: *d*=−0.29, CI −0.72 to 0.14; T3: *d*=−0.32, CI −0.77 to 0.14). Findings from the sensitivity analysis (including 74‐77 participants across outcomes; see Table S4 in Multimedia Appendix 1) closely mirrored those of the intention-to-treat analysis (Table 3) for both the primary and secondary outcomes.

### Use, Helpfulness, and Negative Experiences

Of the 61 adolescents belonging to the intervention group, 46 participants responded to the app evaluation questions in the 2-month follow-up. The frequency of app use reported in the survey varied, with most participants reporting using it multiple times per week (8/46, 17%), sometimes per week (17/46, 37%), or less than once per week (18/46, 39%), while 1 participant reported daily use and 2 participants disclosed not using the app. Objective usage data similarly indicated regular engagement with the app during the first 2 months. During this period, participants completed an average of 9.1 engaged sessions per active user, with an average engagement time of 1 hour and 2 minutes per active user and an average session duration of 18 minutes and 19 seconds.

In the evaluation, most app users reported feeling helped by the app in a variety of ways (Table 4). For instance, a substantial portion of users reported feeling helped completely or to a large extent in understanding grief (mean 2.4‐2.6, SD 0.8), better managing grief and emotions (mean 2.6, SD 0.8), and feeling more capable of positively impacting one’s well-being (mean 2.8, SD 0.8). Notably, although a portion of users did not feel helped at all in terms of facilitating communication (mean 2.0, SD 0.8) and social support (mean 2.1, SD 0.8), the majority felt helped to some extent also in these areas.

When asked about negative experiences with the app, most participants (n=36) reported none, while 9/45 (20%) participants did. Analysis of the free-text responses for these 9 adolescents revealed that for 6 participants, these negative events related to how the app triggered sadness related to their loss. Similarly, 6 participants reported that the app evoked thoughts and emotions connected to grief; however, 3 of them also emphasized that revisiting and making room for these emotions was necessary and experienced as positive.

**Table 4.** Survey responses on the helpfulness of the Alba app (N=46).

Has the Alba app helped you…	4 (yes, completely), n (%)	3 (to a large extent), n (%)	2 (to some extent), n (%)	1 (no, not at all), n (%)	Mean score (SD)
Understand what grief is?	4 (8.7)	21 (45.7)	20 (43.5)	1 (2.2)	2.6 (0.7)
Identify common grief reactions in yourself?	2 (4.4)	17 (37.0)	25 (54.4)	2 (4.4)	2.4 (0.7)
Better understand your grief reactions?	4 (8.7)	16 (34.8)	22 (47.8)	4 (8.7)	2.4 (0.8)
Differentiate between common grief reactions and more severe reactions, which one may require help with?	4 (8.7)	18 (39.1)	20 (43.5)	4 (8.7)	2.5 (0.8)
Better handle your emotions and your grief?	4 (8.7)	23 (50.0)	15 (32.6)	4 (8.7)	2.6 (0.8)
Feel that there is something you can do to feel better?	8 (17.4)	25 (54.4)	11 (23.9)	2 (4.4)	2.8 (0.8)
To talk to others about your emotions and your grief?	1 (2.2)	11 (23.9)	19 (41.3)	15 (32.6)	2.0 (0.8)
Seek support and help when you need it?	1 (2.2)	14 (30.4)	18 (39.1)	13 (28.3)	2.1 (0.8)

## Discussion

### Principal Findings

This study examined the short- and long-term effects of the self-management mobile app Alba – Youth in Grief on mental health outcomes among bereaved adolescents, alongside user-reported helpfulness and negative experiences of using the app. Overall, the Alba app yielded greater reductions in grief reactions and symptoms of prolonged grief, posttraumatic stress, and depression compared with an active control condition, with the strongest effects observed at the one-year follow-up. No effects were observed for personal growth. Most adolescents reported the app as helpful, particularly for understanding grief, managing emotion, and strengthening self-efficacy, while only a minority reported negative experiences of using the app.

For the primary outcome, prolonged grief, no significant app effects were observed at 2 or 6 months; however, a moderate effect emerged at 12 months. This pattern resembles findings from evaluations of the adult My Grief app, which demonstrated small to moderate effects on prolonged grief across follow-ups when compared with a waitlist control, with effects likewise largest at one year [23,24]. A similar trajectory has been reported in in-person CBT treatments for PGD in children and adolescents, where small short-term effects increased to moderate levels at long-term follow-up when compared with an active control condition [52]. This delayed effect may reflect the preventive and low-intensity nature of the intervention, in which adolescents engaged with the app freely without guidance regarding frequency or patterns of use. Given that preventive grief interventions for bereaved adolescents generally demonstrate relatively small effects beyond the natural course of grief over time [14], meaningful between-group differences in prolonged grief symptoms may require sustained engagement and longer follow-up periods to emerge. Additionally, adolescents’ engagement with mHealth interventions has been shown to vary over time [22], which may further contribute to the delayed intervention effects. In contrast, unguided internet-delivered CBT was unable to demonstrate significant effects on PGD when compared to a waitlist control in a pilot trial [20], and a meta-analysis indicates that preventive grief interventions generally only yield small effects under controlled conditions [14]. Given that treatment interventions and waitlist-controlled designs typically produce larger effect sizes than preventive interventions evaluated against active controls [14], the moderate long-term effects observed for Alba on prolonged grief symptoms appear promising in relation to the existing literature.

Regarding secondary outcomes, significant between-group differences in change from baseline were observed for grief reactions, posttraumatic stress, and depressive symptoms at 2 and 12 months, but not at 6 months. Improvements in the control group during the mid-follow-up period reduced between-group differences at 6 months, suggesting some degree of natural recovery or benefits associated with the control intervention. Overall, Alba demonstrated small to moderate effects on grief reactions, moderate effects on posttraumatic stress symptoms, and small effects on depressive symptoms. This pattern closely mirrors findings from My Grief, which reported small to moderate effects on posttraumatic stress symptoms and smaller effects on depression [23,24]. Notably, these findings diverge from earlier preventive grief interventions, which have generally been unable to demonstrate effects on posttraumatic stress symptoms or depression in controlled designs [14]. Instead, the trajectory of effects in our study, particularly the strengthening of effects over time, more closely resembles that observed in CBT-based treatment interventions for bereaved adolescents [20,52]. This resemblance may reflect the CBT-informed content of Alba, as well as the relatively elevated symptom levels in the present sample, given that interventions tend to yield larger effects in samples characterized by higher levels of distress [14]. This similarity also extends to the magnitude of effects. Although in-person CBT did not demonstrate short-term effects on depression or posttraumatic stress, it showed small long-term effects on depression and moderate long-term effects on posttraumatic stress symptoms when compared with an active control condition, similar to our study [52].

While our findings, consistent with previous research, suggest that intervention effects may consolidate over time, the observed trajectory warrants consideration. Notably, app effects were not statistically significant at 6 months, despite being evident both at earlier and later time points. One explanation may relate to limited or declining app engagement following initial use, a pattern observed in mHealth interventions for adolescents [22]. Although objective usage data indicated that participants engaged with Alba repeatedly during the first 2 months, averaging approximately 9 engaged sessions and just over one hour of cumulative engagement per active user, engagement may nevertheless have declined thereafter. However, the association between app engagement and well-being outcomes is difficult to interpret. Lower app engagement over time is not necessarily negative, as it may partly reflect reduced support needs following improved well-being. It may also indicate that users have integrated coping strategies and information from the app into their everyday lives, thereby reducing the need for continued app engagement, as described in the narrative evaluation of Alba [27]. Conversely, more frequent app use may in some cases reflect difficulties incorporating the app content into daily routines or a continued need for support.

Another contributing factor to effects solidifying over time may be the emotional demands of engaging with grief-related content, as previously noted in relation to bereavement support interventions [18]. Some adolescents reported that the app elicited sadness and grief-related thoughts; a tendency observed also in the narrative evaluation of Alba [27], where adolescents described the emotional challenge of confronting grief following prolonged reliance on avoidance, and how the app promoted gradual development of more helpful coping strategies. Approaching grief and reducing avoidance in this sense can thus be emotionally demanding [18] and may involve symptom fluctuations before improvements stabilize. Given established links between avoidance and elevated prolonged grief and posttraumatic stress symptoms [10], the emotional discomfort associated with app use may potentially represent an important process contributing to long-term mental health improvements. However, these potential mechanisms were not directly examined in the present study and require further investigation. Importantly, the absence of reports of more severe negative experiences further supports the safety of Alba as a preventive intervention.

Despite overall positive findings, no statistically significant effects were observed for personal growth. Posttraumatic growth has previously been demonstrated among Swedish bereaved adolescents within a support group setting, where small effects were observed over a comparable time frame, and was positively linked to social and professional support, when assessed using the Posttraumatic Growth Inventory [12]. While differences in measurement instruments may partly account for the divergence in findings, the results may also suggest that relational and social components, generally more prominent in in-person and group-based formats, are important for promoting personal growth following bereavement [12]. Nonetheless, adolescents in the narrative evaluation of Alba [27] described internal changes consistent with posttraumatic growth, describing feeling stronger, more empathetic, and increasingly appreciative of loved ones. In this context, the absence of group-level effects may potentially relate to the relatively high levels of distress in the current sample. Prior research has suggested an inverted U-shaped relationship between distress and posttraumatic growth in young people, whereby low distress may not stimulate growth, while high distress may inhibit it, suggesting moderate levels to be most conducive to posttraumatic growth [53,54]. With indications also that reductions in distress may act as a catalyst for subsequent growth [53], it is further possible that posttraumatic growth may emerge later, following symptom alleviation.

The high distress levels observed within the sample are further noteworthy, given that Alba was designed as a preventive intervention and the trial did not have symptom-based inclusion criteria. Most participants reported symptom levels indicative of probable PGD, PTSD, or depression, with the 33%‐40% prevalence of probable PGD exceeding estimates reported in previous studies [8]. This pattern may reflect the particular appeal of mHealth interventions. Given the accessibility, flexibility, and potential for anonymity [19], mHealth apps may serve as a low-threshold alternative for adolescents who are hesitant to engage in in-person or group-based support and who may otherwise delay or avoid help-seeking altogether [55]. Thus, although CBT principles are commonly incorporated into preventive interventions delivered in traditional formats, such as support groups [14], the mHealth format offers additional advantages related to reach, scalability, and potential cost-effectiveness [19], positioning Alba as a valuable complement to existing bereavement support services.

Consistent with symptom outcomes, self-reported helpfulness ratings indicated that adolescent participants perceived Alba to be helpful, particularly for understanding grief, managing emotions, and strengthening self-efficacy. These findings closely parallel evaluations of My Grief, where participants similarly reported enhanced grief understanding, coping, and confidence in managing their well-being [32]. Although the helpfulness areas of improved help-seeking and communication were less strongly endorsed in both studies [32], a substantial proportion of adolescents nevertheless reported benefits in these domains, which is an important finding given the protective potential of emotional expression and social support in bereaved adolescents’ mental health [8,11]. The comparatively lower endorsement of these areas is perhaps unsurprising, as many bereaved adolescents may be reluctant to talk about grief or accept social support, while opportunities for communication are also highly dependent on the broader social environment [1,5]. Thus, lower ratings in these domains may partly reflect different coping preferences or contextual barriers, but may also suggest a need for stronger emphasis on communication and support-seeking strategies in future app development. However, reported gains in self-awareness, adaptive coping, and self-efficacy, observed both in self-reports and the narrative evaluation [27], further suggest that Alba may have the potential to strengthen protective factors relevant to bereaved adolescents’ mental health, although the relationship between these factors and mental health outcomes requires further investigation [8,11].

This study has several limitations, including how the sample consisted primarily of female and Sweden-born adolescents, limiting the generalizability to more diverse populations. In addition, the TGI-K-SR+, used to assess prolonged grief, is a newly developed instrument, and the Swedish translation has not yet undergone extensive psychometric validation. Given that knowledge of how prolonged grief manifests in children and adolescents remains limited and that the measure is largely informed by adult instruments [36], findings related to prolonged grief outcomes should be interpreted cautiously. Similarly, the results for the secondary outcome measures should also be interpreted with caution, as these analyses are exploratory in nature and the current study was not powered to detect effects in these outcomes. Additionally, although LMMs are appropriate under the assumption that data are missing at random, baseline comparisons indicated that noncompleters in the intervention group reported higher posttraumatic stress and depressive symptoms than noncompleters in the control group. If these higher levels persisted at subsequent time points with missing data, this would suggest that missingness may be related to symptom severity at those time points (missing not at random), potentially contributing to an overestimation of intervention effects. Nevertheless, sensitivity analysis based on complete cases yielded comparable results, suggesting that the findings were relatively robust to attrition. Furthermore, the use of other professional support resources during this study was not assessed, and although app interaction data were collected, this will not enable assessment of how the extent and patterns of app use may relate to the observed outcomes. Nonetheless, the current study also demonstrates substantial methodological strengths which, in addition to the randomized controlled design, intention-to-treat approach, and long-term follow-up [14], include satisfactory retention rates and the collaborative approach to app development and trial design, which likely enhanced acceptability and feasibility [25,26]. Future research should, however, examine Alba’s effects in more diverse samples and explore how patterns and intensity of app use translate into reductions in mental health symptoms.

Overall, the findings suggest that Alba is a safe, acceptable, and beneficial mHealth intervention that may serve as an important complement to existing sources of bereavement support. Owing to its flexibility and accessibility, Alba may function as a low-threshold intervention for bereaved adolescents and, although evaluated here only as an individually used intervention, may also be suitable for use within group settings or alongside professional support. For instance, the app could provide support between group sessions or meetings with mental health professionals, and, as observed in the narrative evaluation [27], adolescents’ documentation of grief intensity and emotions within the app may serve as a basis for therapeutic discussions. Exercises focused on memorializing or writing about the deceased may likewise lend themselves to shared group activities and collective reflection.

### Conclusions

To our knowledge, this study is the first to evaluate the short- and long-term effects of a self-management mobile app for bereaved adolescents. The findings suggest that the Alba app yielded greater reductions in grief reactions and symptoms of prolonged grief, posttraumatic stress, and depression compared with an active control group receiving web-based psychoeducation. Overall, the largest effects emerged at long-term follow-up, with predominantly moderate effect sizes observed after 12 months, suggesting that the benefits of app use may strengthen over time. Only a minority of participants reported negative experiences, primarily related to the app eliciting thoughts and emotions connected to their loss. At the same time, most adolescents reported that the app helped them better understand grief and their own grief reactions, manage emotions, and strengthen their sense of self-efficacy, communication, and support-seeking. Together, these findings indicate that Alba may constitute a helpful approach for supporting the mental health of grieving adolescents and underscore its potential as an accessible and scalable source of bereavement support.

## Supplementary material

10.2196/94777 Multimedia Appendix 1 Outputs of the dropout and sensitivity analyses.

10.2196/94777 Checklist 1 CONSORT-eHEALTH V 1.6.1.

## Abbreviations

CBT: cognitive behavioral therapy
*DSM-5*: *Diagnostic and Statistical Manual of Mental Disorders, Fifth Edition*
*DSM-5-TR*: *Diagnostic and Statistical Manual of Mental Disorders, Fifth Edition, Text Revision*
HIBSF-CA: Hogan Inventory for Bereavement–Short Form for Children and Adolescents
*ICD-11*: *International Classification of Diseases, 11th Revision*
LMM: linear mixed model
mHealth: mobile health
PGD: prolonged grief disorder
PTSD: posttraumatic stress disorder
RCT: randomized controlled trial
TGI-K-SR+: Traumatic Grief Inventory–Kids–Self Report+
