# Supplementary material for: Effects and User-Reported Experiences of a Self-Management Mobile Health App for Grieving Adolescents: Randomized Controlled Trial
Source: J Med Internet Res. 2026 Jul 15;28:e94777. doi: 10.2196/94777 (PMC13373461; doi:10.2196/94777)
Supplement: Multimedia Appendix 1 [file jmir-v28-e94777-s001.docx]

**Table S1.** Comparison of primary and secondary outcome variables at baseline between dropouts in the intervention and control group.

| Measure | Group | N | M | SD | *t* | df | *p* |
| --- | --- | --- | --- | --- | --- | --- | --- |
| Prolonged grief | Intervention | 23 | 51.09 | 14.90 | -.12 | 35 | .90 |
|  | Control | 26 | 50.65 | 8.81 |  |  |  |
| Grief | Intervention | 23 | 26.57 | 9.56 | -.91 | 43 | .37 |
|  | Control | 26 | 24.27 | 7.82 |  |  |  |
| Personal growth | Intervention | 23 | 27.78 | 7.87 | 1.84 | 46 | .07 |
|  | Control | 26 | 31.85 | 7.51 |  |  |  |
| Posttraumatic stress | Intervention | 23 | 34.12 | 20.80 | -2.31 | 34 | .03 |
|  | Control | 26 | 22.77 | 11.80 |  |  |  |
| Depression | Intervention | 23 | 12.74 | 7.29 | -2.69 | 35 | .01 |
|  | Control | 26 | 8.94 | 4.38 |  |  |  |

**Table S2.** Comparison of primary and secondary outcome variables at baseline between participants in the intervention group who completed all assessment points and those who did not complete all assessment points.

| Measure | Group | N | M | SD | *t* | df | *p* |
| --- | --- | --- | --- | --- | --- | --- | --- |
| Prolonged grief | Completers | 38 | 51.37 | 10.70 | -.08 | 36 | .94 |
|  | Non-completers | 23 | 51.09 | 14.90 |  |  |  |
| Grief | Completers | 38 | 27.76 | 7.19 | -.52 | 37 | .60 |
|  | Non-completers | 23 | 26.57 | 9.56 |  |  |  |
| Personal growth | Completers | 38 | 34.05 | 8.50 | -2.92 | 49 | .01 |
|  | Non-completers | 23 | 27.78 | 7.87 |  |  |  |
| Posttraumatic stress | Completers | 38 | 28.82 | 20.80 | 1.09 | 34 | .28 |
|  | Non-completers | 23 | 34.13 | 13.60 |  |  |  |
| Depression | Completers | 38 | 10.87 | 6.23 | 1.03 | 41 | .31 |
|  | Non-completers | 23 | 12.74 | 7.29 |  |  |  |

**Table S3.** Comparison of primary and secondary outcome variables at baseline between participants in the control group who completed all assessment points and those who did not complete all assessment points.

| Measure | Group | N | M | SD | *t* | df | *p* |
| --- | --- | --- | --- | --- | --- | --- | --- |
| Prolonged grief | Completers | 39 | 49.72 | 9.98 | .40 | 58 | .69 |
|  | Non-completers | 26 | 50.65 | 8.81 |  |  |  |
| Grief | Completers | 39 | 26.38 | 6.80 | -1.13 | 48 | .27 |
|  | Non-completers | 26 | 24.27 | 7.82 |  |  |  |
| Personal growth | Completers | 39 | 34.41 | 8.25 | -1.30 | 57 | .20 |
|  | Non-completers | 26 | 31.85 | 7.51 |  |  |  |
| Posttraumatic stress | Completers | 39 | 27.38 | 12.70 | -1.50 | 56 | .14 |
|  | Non-completers | 26 | 22.77 | 11.80 |  |  |  |
| Depression | Completers | 39 | 10.28 | 4.97 | -1.92 | 58 | .06 |
|  | Non-completers | 26 | 8.04 | 4.38 |  |  |  |

**Table S4.** Sensitivity analyses (including participants who completed all assessments) LMM results for primary and secondary outcomes. Model estimates represent changes from baseline (T0) and group differences relative to the Control group reference category.

| Outcome | Fixed effect | Estimate (β) | SE | 95% CI | P-value |
| --- | --- | --- | --- | --- | --- |
| Prolonged grief | Intercept | 49.72 | 1.76 | 46.29 to 53.15 | <.001 |
| (n=77) | Condition | 1.65 | 2.50 | -3.23 to 6.53 | .511 |
|  | Time (T0-T1) | -1.92 | 1.37 | -4.58 to .73 | .161 |
|  | Time (T0-T2) | -6.49 | 1.37 | -9.14 to -3.83 | <.001 |
|  | Time (T0-T3) | -6.18 | 1.37 | -8.84 to -3.52 | <.001 |
|  | Condition x T0-T1 | -4.10 | 1.95 | -7.89 to -.32 | .036 |
|  | Condition x T0-T2 | -2.96 | 1.95 | -6.74 to .82 | .130 |
|  | Condition x T0-T3 | -7.24 | 1.95 | -11.02 to -3.46 | <.001 |
| Grief | Intercept | 26.05 | 1.10 | 23.92 to 28.19 | <.001 |
| (n=74) | Condition | 1.51 | 1.55 | -1.51 to 4.54 | .332 |
|  | Time (T0-T1) | -.24 | .91 | -2.00 to 1.52 | .789 |
|  | Time (T0-T2) | -2.43 | .91 | -4.19 to -.67 | .008 |
|  | Time (T0-T3) | -1.54 | .91 | -3.30 to .22 | .090 |
|  | Condition x T0-T1 | -2.51 | 1.28 | -5.00 to -.03 | .051 |
|  | Condition x T0-T2 | -.46 | 1.28 | -2.95 to 2.03 | .720 |
|  | Condition x T0-T3 | -4.30 | 1.28 | -6.78 to -1.81 | <.001 |
| Personal growth | Intercept | 34.49 | 1.43 | 31.70 to 37.27 | <.001 |
| (n=74) | Condition | -.03 | 2.02 | -3.97 to 3.91 | .989 |
|  | Time (T0-T1) | -.49 | 1.12 | -2.66 to 1.69 | .665 |
|  | Time (T0-T2) | .73 | 1.12 | -1.45 to 2.90 | .515 |
|  | Time (T0-T3) | .41 | 1.12 | -1.77 to 2.58 | .718 |
|  | Condition x T0-T1 | 1.84 | 1.58 | -1.24 to 4.91 | .247 |
|  | Condition x T0-T2 | 2.51 | 1.58 | -.56 to 5.59 | .114 |
|  | Condition x T0-T3 | 2.86 | 1.58 | -.21 to 5.94 | .072 |
| Posttraumatic stress | Intercept | 26.95 | 2.24 | 22.58 to 31.31 | <.001 |
| (n=74) | Condition | 1.41 | 3.17 | -4.77 to 7.58 | .658 |
|  | Time (T0-T1) | .62 | 1.77 | -2.81 to 4.05 | .725 |
|  | Time (T0-T2) | -4.65 | 1.77 | -8.08 to -1.22 | .009 |
|  | Time (T0-T3) | -4.11 | 1.77 | -7.54 to -.68 | .021 |
|  | Condition x T0-T1 | -5.73 | 2.50 | -10.58 to -.88 | .023 |
|  | Condition x T0-T2 | -2.43 | 2.50 | -7.28 to 2.42 | .331 |
|  | Condition x T0-T3 | -9.11 | 2.50 | -13.96 to -4.26 | <.001 |
| Depression | Intercept | 10.28 | .85 | 8.63 to 11.94 | <.001 |
| (n=77) | Condition | .59 | 1.21 | -1.77 to 2.94 | .629 |
|  | Time (T0-T1) | -.13 | .73 | -1.54 to 1.28 | .860 |
|  | Time (T0-T2) | -1.72 | .73 | -3.13 to -.31 | .019 |
|  | Time (T0-T3) | -1.79 | .73 | -3.20 to -.39 | .014 |
|  | Condition x T0-T1 | -1.98 | 1.03 | -3.98 to .03 | .057 |
|  | Condition x T0-T2 | -.31 | 1.03 | -2.31 to 1.70 | .766 |
|  | Condition x T0-T3 | -1.99 | 1.03 | -4.00 to .01 | .055 |
